# Disentangling *PTEN*-cooperating tumor suppressor gene networks in cancer

**DOI:** 10.1080/23723556.2017.1325550

**Published:** 2017-05-04

**Authors:** Jorge de la Rosa, Julia Weber, Roland Rad, Allan Bradley, Juan Cadiñanos

**Affiliations:** a The Wellcome Trust Sanger Institute, Wellcome Trust Genome Campus, Hinxton, Cambridgeshire, UK; b Instituto de Medicina Oncológica y Molecular de Asturias (IMOMA), Oviedo, Spain; c Departamento de Bioquímica y Biología Molecular, Facultad de Medicina, Instituto Universitario de Oncología (IUOPA), Universidad de Oviedo, Oviedo, Spain; d Department of Internal Medicine II, Klinikum rechts der Isar, Technische Universität München, München, Germany; e German Cancer Consortium (DKTK), German Cancer Research Center (DKFZ), Heidelberg, Germany

**Keywords:** AKAP13, CELF2, PARD3, prostate cancer, PTEN, Sleeping Beauty, transposon, tumor suppressor gene, WAC, ZBTB20

## Abstract

We have recently performed a whole-body, genome-wide screen in mice using a single-copy inactivating transposon for the identification of *Pten* (phosphatase and tensin homolog)-cooperating tumor suppressor genes (TSGs). We identified known and putative TSGs in multiple cancer types and validated the functional and clinical relevance of several promising candidates for human prostate cancer.

Cancer originates and evolves through the gradual accumulation of genetic/epigenetic alterations in oncogenes and tumor suppressor genes (TSGs). The *PTEN* (phosphatase and tensin homolog) gene is the second most frequently mutated/deleted TSG in human cancer, only after *TP53* (tumor protein p53). As the main antagonist of the phosphatidylinositol 3-kinase (PI3K)-AKT serine/threonine kinase 1 (AKT) oncogenic pathway and a key maintainer of genomic stability, PTEN controls a plethora of cellular processes including metabolism, cell growth, proliferation, and survival.^1^ Although subtle changes in PTEN levels can lead to tumor initiation, lower levels of this protein are linked to more advanced disease, and accompanying mutations in other genes are required for full malignancy.^2^ Identifying these *PTEN*-cooperating TSG networks is a major goal for the understanding of the molecular mechanisms involved in cancer progression and the design of combinatorial therapies to treat PTEN-deficient cancers.^3^

Insertional mutagenesis screens complement human genome sequencing-based approaches for elucidating the genetic forces driving cancer progression.^4,5^ By coupling *Pten*-disruption to mobilization of a *Sleeping Beauty* inactivating transposon within each cell, we have recently performed a novel genome-wide survey for *Pten*-cooperating TSGs in mice.^6^ The transposon, targeted to the *Pten* locus, carries a critical exon of this gene when it is mobilized, leading to *Pten* inactivation and subsequent generation of an additional mutation when randomly reinserted into the genome (Fig. 1). Moreover, increased transposon mutation rate was achieved in a subset of mice by introducing a transgene with additional copies of an inactivating transposon. Based on this innovation, we have identified sets of hundreds of known and novel cancer genes involved in prostate, breast, and skin cancer, all of them predicted to behave as TSGs.^6^ We then focused on prostate cancer, for which PTEN relevance is well documented, and validated the implication of several of the genes identified for the progression of the disease in humans.^6^

**Figure 1.**
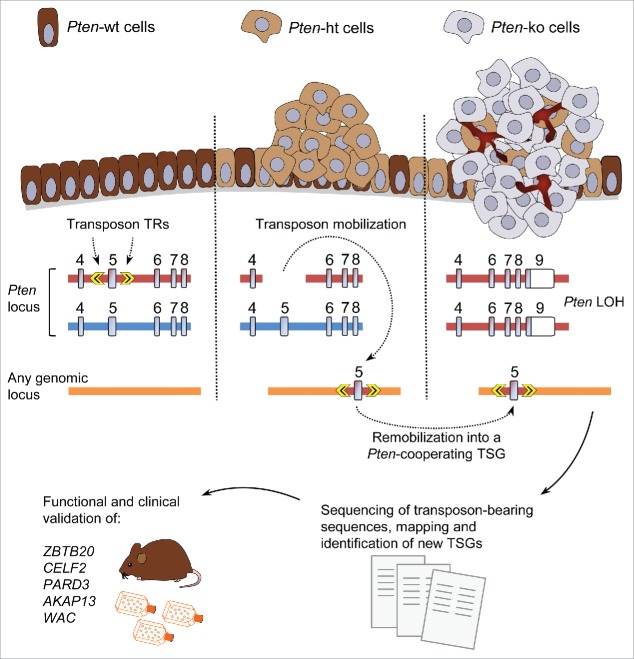
Transposon-based screen for identifying *Pten*-cooperating tumor suppressors in cancer. Mice carry a *Pten* allele where the exon 5 (encoding the phosphatase domain) is flanked by the terminal repeats (TRs) of the *Sleeping Beauty* transposon (top left). This allele functions normally, but it becomes inactivated upon mobilization of the transposon, which subsequently can be reinserted elsewhere in the genome, potentially generating an additional loss-of-function mutation (top middle). Loss-of-heterozygosity (LOH) can lead to inactivation of the second *Pten* allele and/or of the additional, *Pten*-cooperating, mutations (top right). Sequencing and mapping of transposon insertion sites allow identification of targeted genes. Final cancer gene lists are generated with those loci hit by transposition significantly more often than predicted by chance across several tumors. Genes of interest can then be selected for further functional and clinical validation (bottom). TSG, tumor suppressor gene; wt, wild-type; ht, heterozygous; ko, knockout; *Pten*, phosphatase and tensin homolog; *ZBTB20*, zinc finger and BTB domain-containing 20; *CELF2*, CUGBP, Elav-like family member 2; *AKAP13*, A-kinase anchor protein 13; *PARD3*, Par-3 family cell polarity regulator; *WAC*, WW domain-containing adaptor with coiled coil.

Prostate cancer is the most common malignancy in men and the second leading cause of male cancer deaths in the Western world.^7^ Nearly 50% of primary and almost 100% of metastatic prostate tumors have genetic alterations in the PI3K-AKT signaling pathway, mostly through loss of *PTEN*.^8^ However, while some of these tumors progress slowly, others rapidly spread beyond the site of origin and metastasize, implying that genetic alterations beyond this pathway may account for such different behaviors.^7^ Finding genetic markers able to distinguish indolent from aggressive disease represents one of the current unmet challenges.

Transposon integration analysis on 127 prostate tumors led us to the identification of over a hundred genes potentially associated to cancer progression. Cross-comparison with human cancer data sets supported the relevance of these genes for *PTEN*-cooperating human prostate tumor suppression as they are significantly enriched in (1) known and putative human cancer genes, (2) genes whose mRNA expression levels decline concomitantly with those of *PTEN* in human prostate cancer samples, and (3) genes frequently inactivated by homozygous deletion in human prostate cancer.^6^ Among them, those encoding chromatin/histone modifiers and involved in RNA metabolic processes (RNA stability, splicing, and transcriptional regulation) are strongly overrepresented, followed by those implicated in ubiquitin-mediated proteolysis (mainly E3 ligases). Interestingly, some of these genes have been described previously to be altered in human prostate cancer through different mechanisms, including mutation (*ARID1A, KDM6A, MLL1, MLL5*, and *MAGI3*), copy-number variation (*ETV6* and *FOXP1*), gene fusion (*TBL1XR1, FUBP1*, and *EPB41*), transcriptional dysregulation (*MEIS1* and *PBX1*), or single nucleotide polymorphism (*RASA1*).^8,9^ This shows the potential of transposon mutagenesis screens to identify a diversity of cancer genes that otherwise require multiple methodological approaches to be pinpointed. Moreover, for several of these genes, our results represent the first piece of biologic evidence for their tumor suppressive role in prostate cancer.^6^

We selected 5 of these genes, ranking among the top 20 most frequently hit by transposition, for further validation. These genes encode the transcription factor ZBTB20 (zinc finger and BTB domain-containing 20), the RNA-binding factor CELF2 (CUGBP, Elav-like family member 2), the controller of cell polarity PARD3 (Par-3 family cell polarity regulator), the scaffold protein AKAP13 (A-kinase anchor protein 13), and the autophagy regulator WAC (WW domain-containing adaptor with coiled coil). We showed that co-silencing the expression of *PTEN* and each of these five genes increased the invasive potential of two immortalized but nontransformed human prostate cell lines.^6^ Moreover, the analysis of their transcriptomic profiling upon co-silencing conditions revealed rewiring of known oncogenic pathways important for prostate cancer progression.^6^ Additionally, we found that each one of these genes is co-downregulated with *PTEN* in primary and metastatic samples, and that patients with tumors expressing low levels of them have worse prognoses, with shorter times to recurrence.^6^ Finally, the generation of mice with prostate-specific heterozygous or homozygous deletion of *Wac* in a*Pten-*deficient background demonstrated that the function of *Wac* in cancer is gene-dose-dependent, as its partial inactivation promotes cancer, but its complete loss constrains tumor growth.^6^ Although downstream analyses are needed to clarify this phenomenon of obligate haploinsufficiency, Wac-mediated regulatory mechanisms of autophagy might be the underlying cause, as autophagy has been shown to exert opposite roles in cancer, depending on the tissue context and its intensity.^10^ Altogether, the genes identified through this screen could not only be used as markers for prognosis and staging purposes, but they may also inspire new anticancer therapies.

Globally, the new mouse model developed in our study provides a very useful tool to disentangle the crosstalk mechanisms between PTEN, a key signaling node in cancer, and previously unknown TSG networks. Thus, cross-comparing the catalog of genes compiled in this screen with previous lists of genes generated through next-generation sequencing of human cancer genomes helps pinpoint the actual drivers of cancer progression, which can be then pursued for downstream analysis.
